#  The Effect of Smartphone App–Based Interventions for Patients With Hypertension: Systematic Review and Meta-Analysis

**DOI:** 10.2196/21759

**Published:** 2020-10-19

**Authors:** Hongxuan Xu, Huanyu Long

**Affiliations:** 1 The Key Laboratory of Geriatrics, Beijing Institute of Geriatrics, Beijing Hospital National Center of Gerontology, National Health Commission Institute of Geriatric Medicine, Chinese Academy of Medical Sciences Beijing China

**Keywords:** hypertension, smartphone, blood pressure, mobile, lifestyle, adherence, smartphone app, medication adherence

## Abstract

**Background:**

Hypertension is a major cause of cardiovascular disease, which is the leading cause of premature death. People with hypertension who do not comply with recommended treatment strategies have a higher risk of heart attacks and strokes, leading to hospitalization and consequently greater health care costs. The smartphone, which is now ubiquitous, offers a convenient tool to aid in the treatment of hypertension through the use of apps targeting lifestyle management, and such app-based interventions have shown promising results. In particular, recent evidence has shown the feasibility, acceptability, and success of digital interventions in changing the behavior of people with chronic conditions.

**Objective:**

The aim of this study was to systematically compile available evidence to determine the overall effect of smartphone apps on blood pressure control, medication adherence, and lifestyle changes for people with hypertension.

**Methods:**

This systematic review was conducted in accordance with the Preferred Reporting Items for Systematic Reviews and Meta-Analysis (PRISMA) statement guidelines. Databases were searched to identify randomized controlled trials related to the influence of an app-based intervention in people with hypertension. Data extracted from the included studies were subjected to a meta-analysis to compare the effects of the smartphone app intervention to a control.

**Results:**

Eight studies with a total of 1657 participants fulfilled the inclusion criteria. Pooled analysis of 6 studies assessing systolic blood pressure showed a significant overall effect in favor of the smartphone intervention (weighted mean difference –2.28, 95% CI –3.90-0.66). Pooled analysis of studies assessing medication adherence demonstrated a significant effect (*P*<.001) in favor of the intervention group (standard mean difference 0.38, 95% CI 0.26-0.50) with low heterogeneity (I^2^=0%). No difference between groups was demonstrated with respect to physical activity.

**Conclusions:**

A smartphone intervention leads to a reduction in blood pressure and an increase in medication adherence for people with hypertension. Future research should focus on the effect of behavior coaching apps on medication adherence, lifestyle change, and blood pressure reduction.

## Introduction

Hypertension, or high blood pressure, is generally defined according to a systolic blood pressure (SBP) reading above 130/140 mmHg and/or diastolic blood pressure (DBP) above 80/90 mmHg. Hypertension is a major cause of cardiovascular disease, which is the leading cause of premature death [1]. Hypertension affects approximately 244.5 million adults in China, only 15.3% of whom have their condition under control [2]. People with hypertension who do not comply with recommended treatment strategies have a higher risk of heart attacks and strokes, leading them to be hospitalized and left with greater health care costs [3]. Although faithfully taking prescribed medication and following suggested lifestyle changes can lead to a dramatic improvement in blood pressure, few people actually follow their doctor’s advice, and thus fail to control their hypertension, leading to high rates of mortality and disability from heart conditions and other vascular diseases [4].

Self-measured blood pressure (SMBP) is believed to improve medication adherence, and is now a common intervention for hypertension management [5,6]. Lifestyle changes such as dietary sodium restriction, weight loss, and aerobic exercise can substantially decrease blood pressure [7]. In addition, the smartphone, which is found everywhere, offers a convenient tool to aid in the treatment of hypertension through the use of apps targeting lifestyle management, which have been showing promising results [8,9]. Recent evidence demonstrates the feasibility, acceptability, and success of digital interventions in changing the behavior of people with chronic conditions [10-17]. Getting one’s hypertension under control may involve many lifestyle and behavioral changes. We hypothesized that smartphone apps combined with regular blood pressure monitoring and digital behavior change interventions may be more effective than currently employed hypertension management strategies.

Functions of such apps include a reminder to take one’s medication, tracking a biometric result, education and motivation, and individualized coaching based on measured values and nonpharmaceutical behaviors. A large number of apps for medication adherence have become available in the last few years [18].

The primary objective of this systematic review and meta-analysis was to analyze the literature to determine the effect of smartphone apps on blood pressure control, medication adherence, and lifestyle changes. Only studies using stand-alone smartphone apps were included in the meta-analysis. Smartphone interventions that are not based on an app were excluded due to not conforming to our primary objective.

## Methods

### Search Strategy

This systematic review was conducted in accordance with the Preferred Reporting Items for Systematic Reviews and Meta-Analysis (PRISMA) statement guidelines [19].

We carried out a keyword search using the terms “smartphone,” “hypertension,” and “randomized controlled trials.” The Ovid MEDLINE, EMBASE, PubMed, and Cochrane Library databases were searched from the start date of May 14, 2020. These databases were searched using a combination of subject headings (such as Medical Subject Headings) and filters (such as “RCT”) when available. We also reviewed the references of included studies to identify additional pertinent studies. We imposed no language or time restriction.

### Inclusion and Exclusion Criteria

Two reviewers independently assessed the records identified from the search for eligibility. Any discrepancies were resolved by consensus. We included any randomized controlled trials comparing smartphone apps–based hypertension management versus usual care or SMBP in adult primary hypertension patients. The target population was adults (aged 18 years and above) with hypertension (as defined by the authors). The outcome had to be objectively measured blood pressure changes. We accepted any duration of intervention.

We excluded studies of patients with confounding chronic conditions such as chronic kidney disease or diabetes mellitus, and those with missing key data. A protocol was developed prior to commencing this review on PROSPERO (CRD42020140926).

### Study Quality

Study quality was assessed by the two authors based on the seven domains defined by the Cochrane Collaboration tool for assessing risk of bias [20]: (1) random sequence

generation; (2) allocation concealment; (3) blinding of participants and personnel; (4) blinding of outcome assessment; (5) incomplete outcome data; (6) selective reporting; and (7) other biases, including baseline imbalance, early stopping, and bias due to vested financial interest or academic bias.

Potential publication bias across studies was assessed using a funnel plot.

### Data Extraction

One author (XX) extracted all data and both authors (XX and LY) reviewed the data for accuracy. The following data were collected: (1) country, duration of the trial, date of publication; (2) numbers of individuals included, inclusion criteria, exclusion criteria; (3) intervention, concomitant intervention; and (4) systolic/diastolic blood pressure change and behavior change (physical activity and medication adherence were the only two behaviors consistently reported in the existing literature).

### Data Synthesis

#### Meta-Analyses

Meta-analysis was performed with Revman 5.3. We used a random-effects model and calculated the weighted mean difference (WMD) to generate pooled estimates of SBP and DBP changes; a random-effects model and standard mean difference (SMD) were used to calculate the intervention effects of medication adherence and physical activity across studies. We calculated the standard deviation using an assumption of a 0.5 correlation for studies that did not report the standard deviation of the mean of change, following the Cochrane Handbook for Systematic Reviews of Interventions [20]. The I^2^ statistic was used to assess the degree of statistical heterogeneity.

Blood pressure changes were divided into two subgroups based on the type of intervention.

#### Trial Sequential Analysis

Trial sequential analysis is a methodology that considers how much information is needed to anticipate a specific required information size [21]. We used the TSA program version 0.9.5.10 Beta (Copenhagen Trial Unit) to adjust the CIs due to sparse data and repetitive testing of cumulative data, and to calculate the required information size. If the cumulative Z-curve crosses a trial sequential monitoring boundary or enters the futility area, it can be concluded that a sufficient level of evidence may have been reached. Conversely, the conclusion evidence is insufficient if the Z-curve does not cross any boundaries. The required information size was calculated based on autogenerated empirical data per input data. We performed the trial sequential analysis at the level of an overall 5% risk of type I error and a power of 20%.

#### Sensitivity Analysis

We conducted a posthoc sensitivity analysis to assess the impact of the potential reporting bias of trials with small sample sizes (N<60).

## Results

### Included Studies

**Figure 1:**
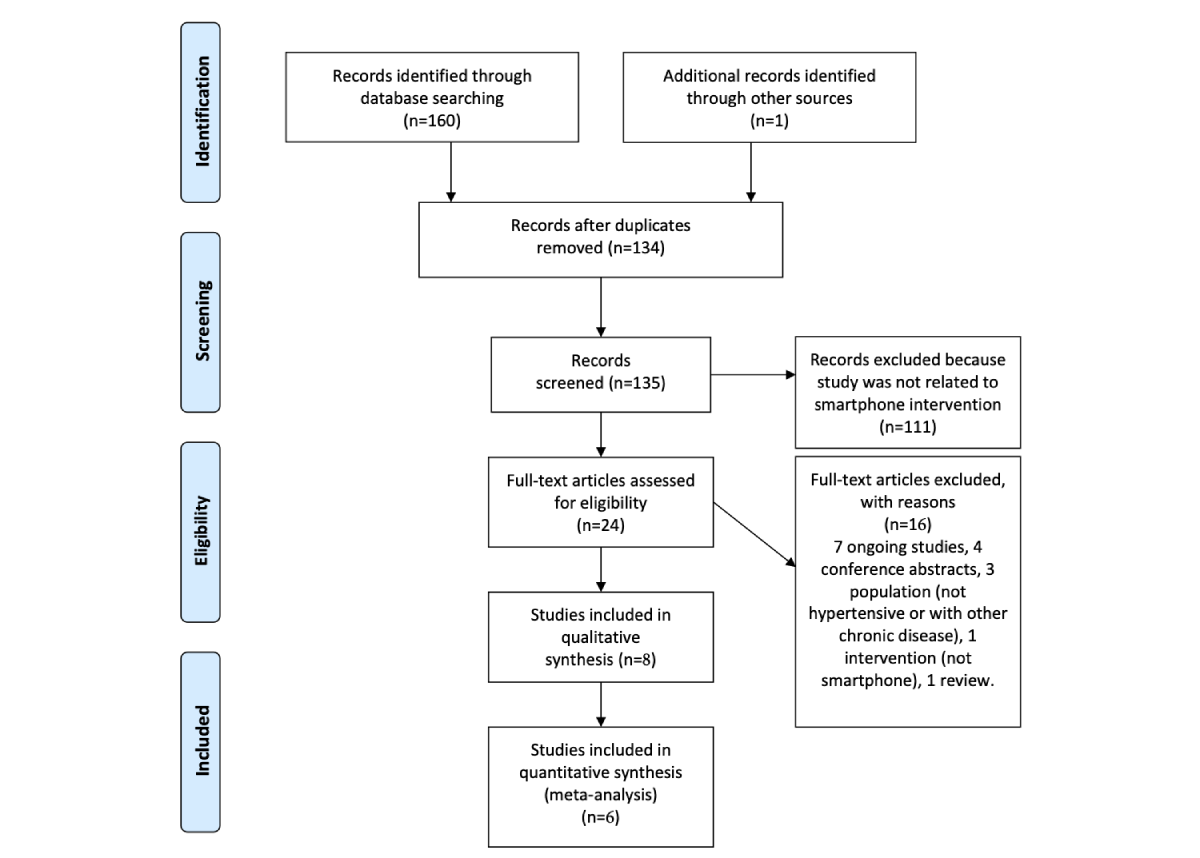
Preferred Reporting Items for Systematic Reviews and Meta-Analyses (PRISMA) flowchart of study selection.

Eight studies [22-29] with a total of 1657 participants fulfilled the inclusion criteria; based on the results of sensitivity analysis, two studies [22,23] were excluded from the meta-analyses (Figure 1). SBP was assessed in 7 studies, DBP was assessed in 6 studies, medication adherence was assessed in 6 studies, and lifestyle changes were assessed in 2 studies. Characteristics of the included studies are summarized in Table 1.

The most common functions of mobile health apps were recording blood pressure, medication reminder, and abnormal values warning. Patient education or health recommendations were reported in 3 studies [23,25,29].

The follow-up period ranged from 6 weeks to 18 months, with a median of 6 months. Over 90% of participants were available for outcome assessment.

**Table 1:** Characteristics of included studies.

Reference	Intervention	Control	Duration	Intervention protocol	Outcomes measured
Persell et al [29]	Hypertension Coaching App, SMBP^a^ (n=144)	BP^b^ tracking app, SMBP (n=152).	6 months	Record (automatically sync) BP daily for the first week, then weekly thereafter, along with reminder, encouragement, and education	BP
Ghezeljeh et al [23]	Social network self-management education (n=25)	Regular routine education (n=25)	6 weeks	Online education weekly	MA^c^
Morawski et al [26]	Medisafe app, SMBP (n=209)	None (n=202)	12 weeks	Record BP, reminder	BP
Logan et al [24]	Telemonitoring Self-Care Support System (n=55)	SMBP (n=55)	12 months	Record BP twice a week and twice in the evening	BP
Gong et al [28]	Yan Fu app, SMBP (n=225)	SMBP, record BP on paper (n=218)	6 months	Record BP at least once daily, reminder	BP, MA
Chandler et al [22]	SMASH app, SMBP (n=28)	enhanced standard care (n=26)	9 months	Record (automatically sync) BP every 3 days in the morning and evening, feedback	BP, MA
Kim et al [25]	app, SMBP, online disease management program (n=52)	usual care, online disease management program (n=43)	6 months	Record BP 3 times a week, 2 measurements per day, health recommendations, reminder	BP, MA
Márquez Contreras et al [27]	ALERHTA app (n=73)	usual care (n=75)	6/18 months	Record BP, reminder	BP, MA

^a^SMBP: self-measured blood pressure.

^b^BP: blood pressure.

^c^MA: medication adherence.

### Risk of Bias

One study was judged to have a low risk of bias [24]. One study was judged to have a high risk of bias at one domain [29]. Results were unclear for the remaining 6 studies, mainly due to lack of detail of performance bias and selection bias (Multimedia Appendix 1).

### Blood Pressure

**Figure 2:**
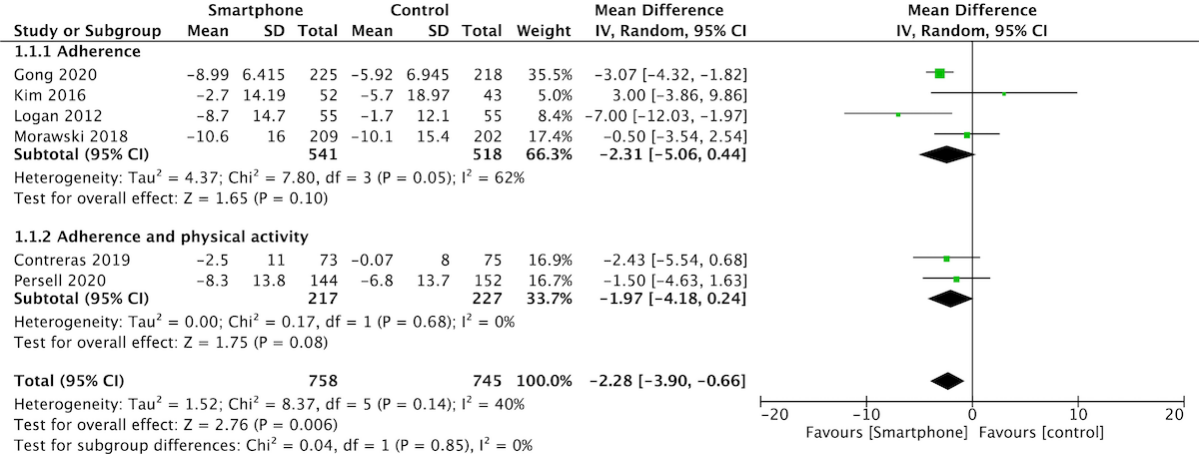
Meta-analysis results and forest plot for the effect of app-based interventions on improvement in systolic blood pressure.

Pooled outcomes of SBP (Figure 2) and DBP (Multimedia Appendix 1) were similar. SBP (WMD –2.28, 95%CI –3.90-0.66; 6 studies) with moderate heterogeneity (I^2^=40%) and DBP (WMD –1.84, 95%CI –3.49 to –0.19; 5 studies) with moderate heterogeneity (I^2^=54%) both showed a significant effect in favor of the intervention (*P*=.006 and *P*=.03, respectively). Blood pressure was significantly reduced in 4 studies (–2.78 mmHg), but was not significantly reduced in 2 studies that included education in the intervention (–0.33 mmHg).

**Figure 3:**
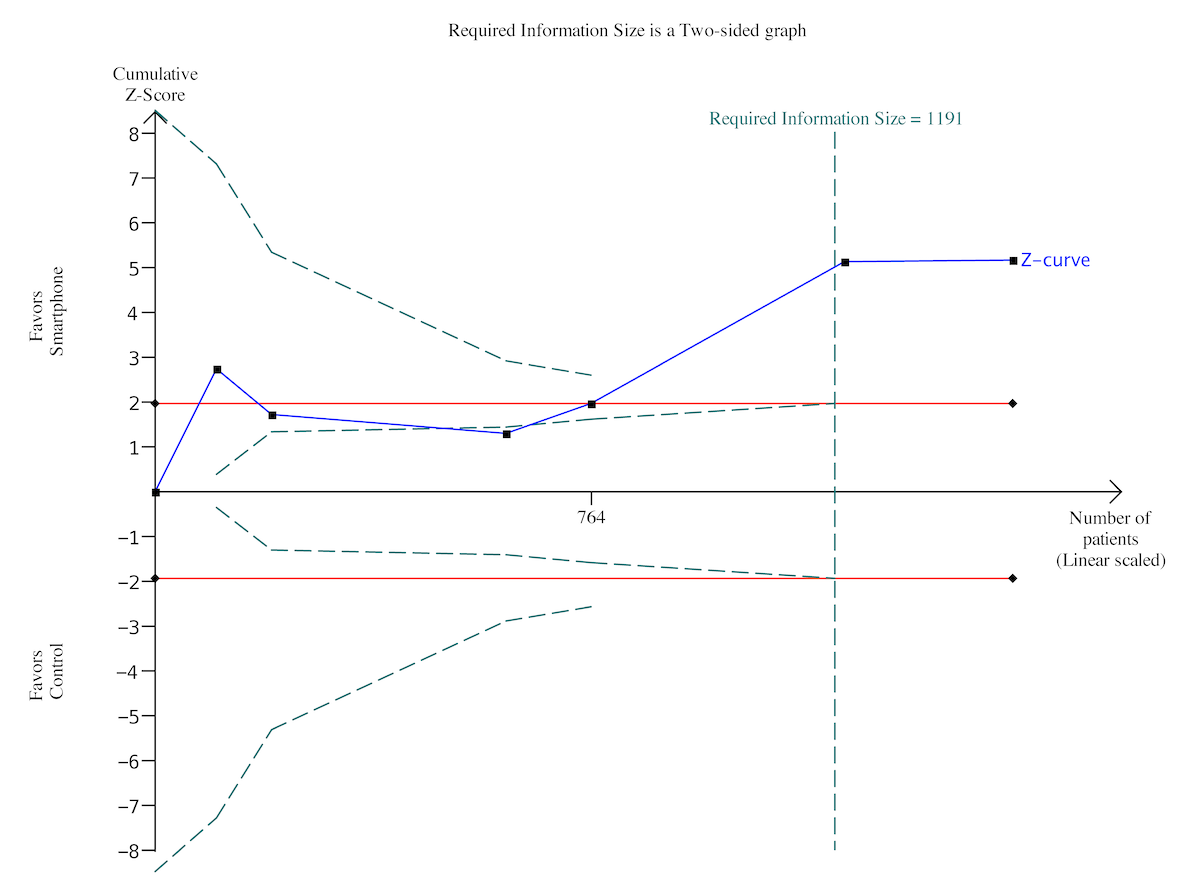
Trial sequential analysis of systolic blood pressure.

Trial sequential analysis showed that the required information size of 20% power had been reached. The certainty of the evidence was high (Figure 3).

### Medication Adherence

**Figure 4:**
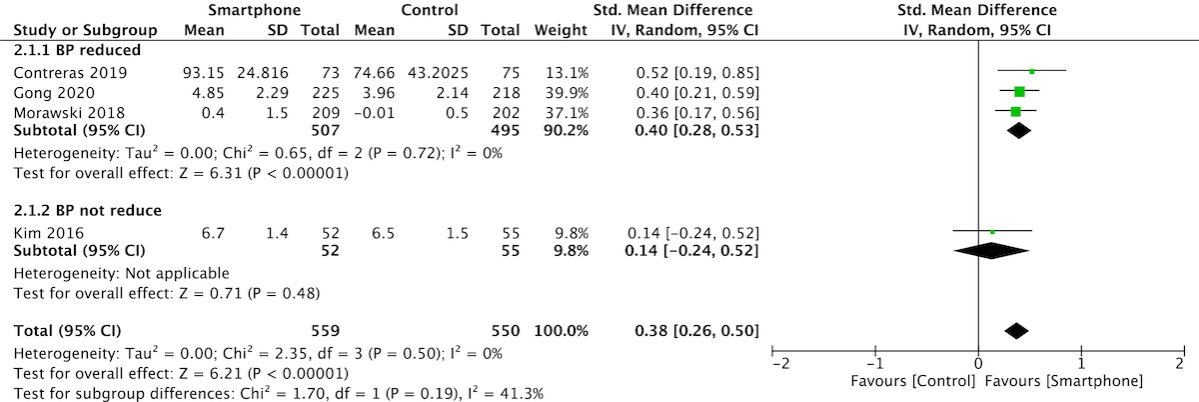
Meta-analysis and forest plot of the effect of the app-based intervention on medication adherence, assessed using the Morisky Medication Adherence Scale-8 item (4 studies).

Four studies assessed medication adherence according to the Morisky Medication Adherence Scale-8 item (Figure 4) [22,25,26,28], one study determined adherence based on the pill count [25], and the other used the Hypertension SM Behavior Questionnaire [23].

Pooled analysis of medication adherence demonstrated a significant effect (*P*<0.0001) in favor of the intervention (SMD 0.38, 95%CI 0.26-0.50) with low heterogeneity (I^2^=0%).

A test for subgroup differences showed an insignificant effect when the studies were grouped (χ^2^=1.70, df=1, I^2^=41.3%).

### Physical Activity

**Figure 5:**
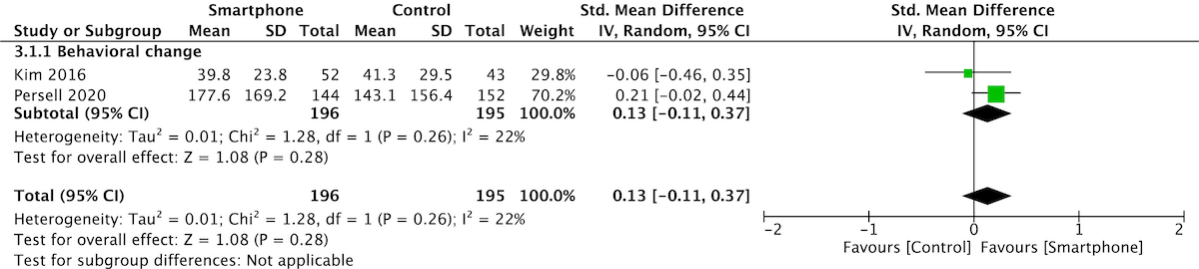
Meta-analysis and forest plot of the effect of the app-based intervention on physical activity (2 studies).

Two studies reported physical activity [25,29]. Pooled analysis did not show a statistically significant effect of the intervention (SMD 0.12, 95%CI –0.13-0.37, *P*=.33; Figure 5). One study showed a significant effect of reducing smoking [25] and one study showed a significant effect of confidence in controlling blood pressure [29].

## Discussion

### Principal Findings

We carried out this review to determine the effect of smartphone apps on the management of hypertension, and a pooled analysis of blood pressure and medication adherence of all studies demonstrated a significant effect in favor of the intervention. However, apps with digital behavior change interventions such as those targeting physical activity demonstrated little effect. The effect size of apps with behavioral instruction functions on blood pressure control was 9 times smaller than that of apps without these functions. Evidence of the impact of smartphone apps on physical activity was insufficient. Pooled analysis of two studies with insignificant blood pressure reduction via promoting physical activity showed a small but insignificant effect in favor of the intervention.

A previous review showed that SMBP alone is not associated with reducing blood pressure, whereas SMBP in conjunction with co-interventions significantly reduced blood pressure [6]. Another review showed that SMBP can lower blood pressure regardless of the number of hypertension-related comorbidities; however, for individuals with conditions such as obesity or stroke, SMBP should be combined with high-intensity co-interventions to effectively reduce blood pressure [30]. Moreover, individuals with chronic conditions using self-management digital interventions feel well cared for and tend to adopt a more active role in their health management [31]. Smartphone apps are able to integrate several co-interventions such as medication adherence, education, and lifestyle recommendations in one device. Our review indicated that SMBP in conjunction with a smartphone app improves medication adherence and reduces blood pressure. A reduction in SBP of 3  mmHg, as observed in intervention groups, would be expected to be associated with an 8% reduction in stroke mortality and a 5% reduction in mortality from coronary heart disease [1]. The observed magnitude of blood pressure reduction would have a significant impact on clinical practice considering the huge population worldwide suffering from hypertension.

A small minority of the apps included in our review incorporated educational functions, and the effect of these functions on blood pressure was insignificant. Available data on medication adherence of these studies was even more rare. The only study that reported medication adherence demonstrated an insignificant result. Yeung et al [32] found that in a low health literacy patient population, the use of educational tools such as flash cards and online videos significantly improved medication adherence in diabetes, hypertension, and heart failure patients. This study demonstrated that the successful education of patients regarding their medication use can significantly improve medication adherence. Medication nonadherence is estimated to cost the health care system US $5824 annually per person among patients with hypertension [33]. Low adherence to antihypertension therapy among hospitalized patients was associated with increased costs of approximately US $3574 (95% CI US $2897-$4249) per person within a 3-year period [3]. This imposes a huge burden on the health care system and patients.

The lack of economic data such as the cost-effectiveness of smartphone-based interventions in improving medication adherence in patients with chronic health conditions highlights the need for further research to understand their role in cost savings while simultaneously improving medication adherence and health outcomes [34,35].

We have reason to believe that smartphone apps are feasible in general practice. Health care professionals should regard smartphone apps as potential tools for patients with hypertension to optimize management [36]. To better utilize these apps, several barriers need to be overcome, such as the generational difference in the propensity to use digital devices, lack of knowledge of available apps, ease of use of apps by the elderly, and privacy and data safety issues [36].

In our review, physical activity yielded an insignificant result. It is critical to discover how to usefully conceptualize and operationalize engagement with digital behavior change interventions. For instance, standardized terminology and measurement techniques will ensure more rapid advances in understanding engagement with digital behavior change interventions and developing methods to improve them [37]. The choice of app may be influenced by its immediate look and feel, “social proof,” titles that appear realistic, and multicomponent features. Design features should focus on enhancing motivation, autonomy, personal relevance, and credibility [38-40].

It is vital that health care professionals and patients join together to form an effective and integrative relationship in clinical practice with mobile health apps.

### Limitations

There were relatively few studies included in this meta-analysis. Therefore, the conclusions may be influenced by publication bias and should be regarded as preliminary. We did not include interventions that involved or targeted pediatric patients. The included trials were mainly conducted in North America and East Asia; thus, geographic unevenness may lead to underlying bias. Choice of the population such as excluding patients with mildly elevated blood pressure and different control interventions may also have yielded bias. With the various terms used to describe a digital intervention and its related health care, some studies may have been overlooked. The statistical findings should be interpreted cautiously for potential underlying heterogeneity.

### Conclusion

A smartphone intervention leads to a reduction in blood pressure and an increase in medication adherence. Future research should discover the effect of behavior coaching apps on medication adherence, lifestyle change, and blood pressure reduction.

## Appendix

Multimedia Appendix 1 Risk of bias assessment for the included studies and meta-analysis for the effect of a smartphone app intervention on diastolic blood pressure (DBP) improvement.

## Abbreviations

DBP: diastolic blood pressure
PRISMA: Preferred Reporting Items for Systematic Reviews and Meta-Analysis
SBP: systolic blood pressure
SMBP: self-measuring blood pressure
SMD: standard mean difference
WMD: weighted mean difference
