# Supplementary figures and images for: The Effect of Smartphone App–Based Interventions for Patients With Hypertension: Systematic Review and Meta-Analysis
Source: JMIR Mhealth Uhealth. 2020 Oct 19;8(10):e21759. doi: 10.2196/21759 (PMC7605981; doi:10.2196/21759)

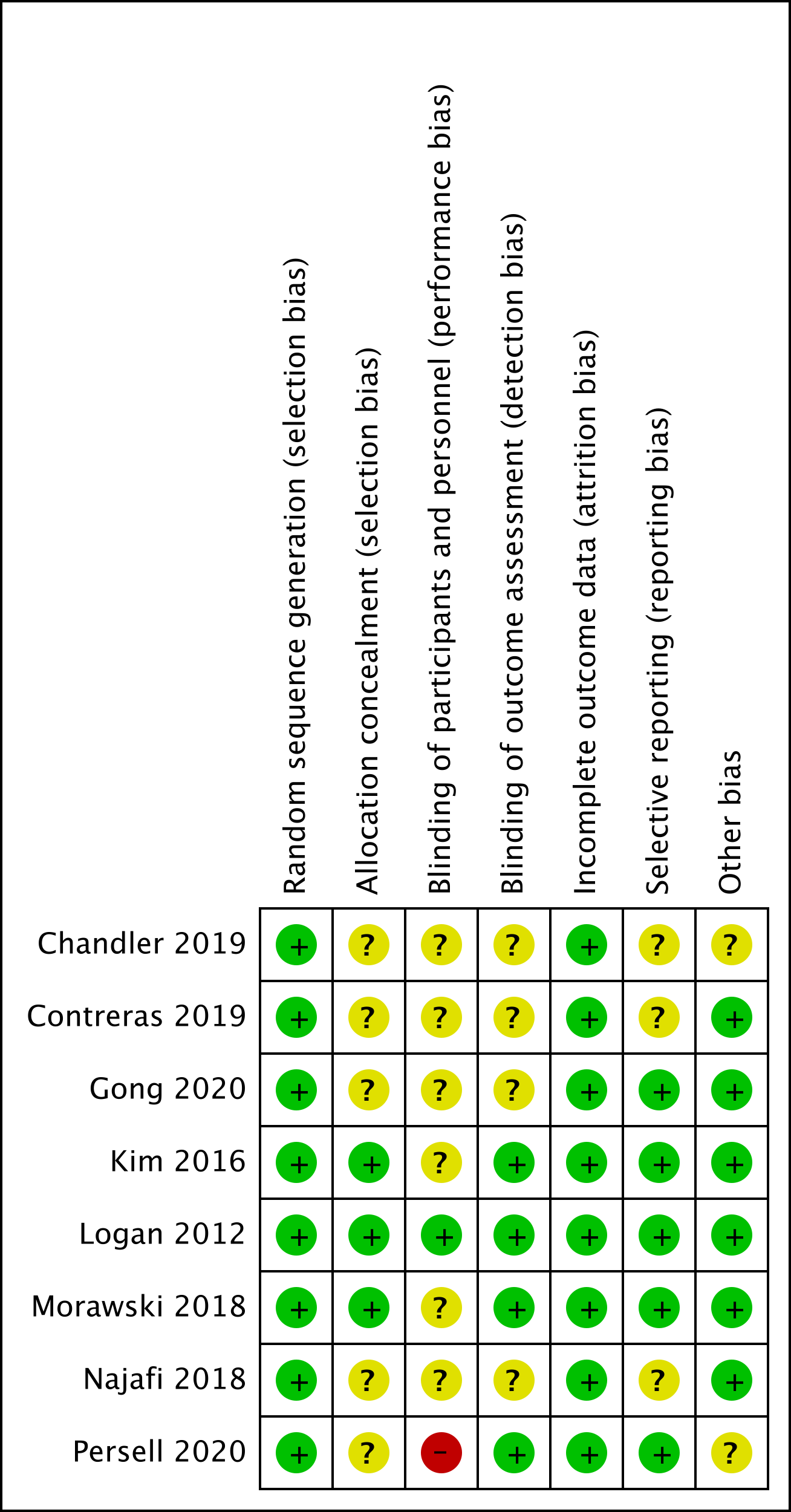

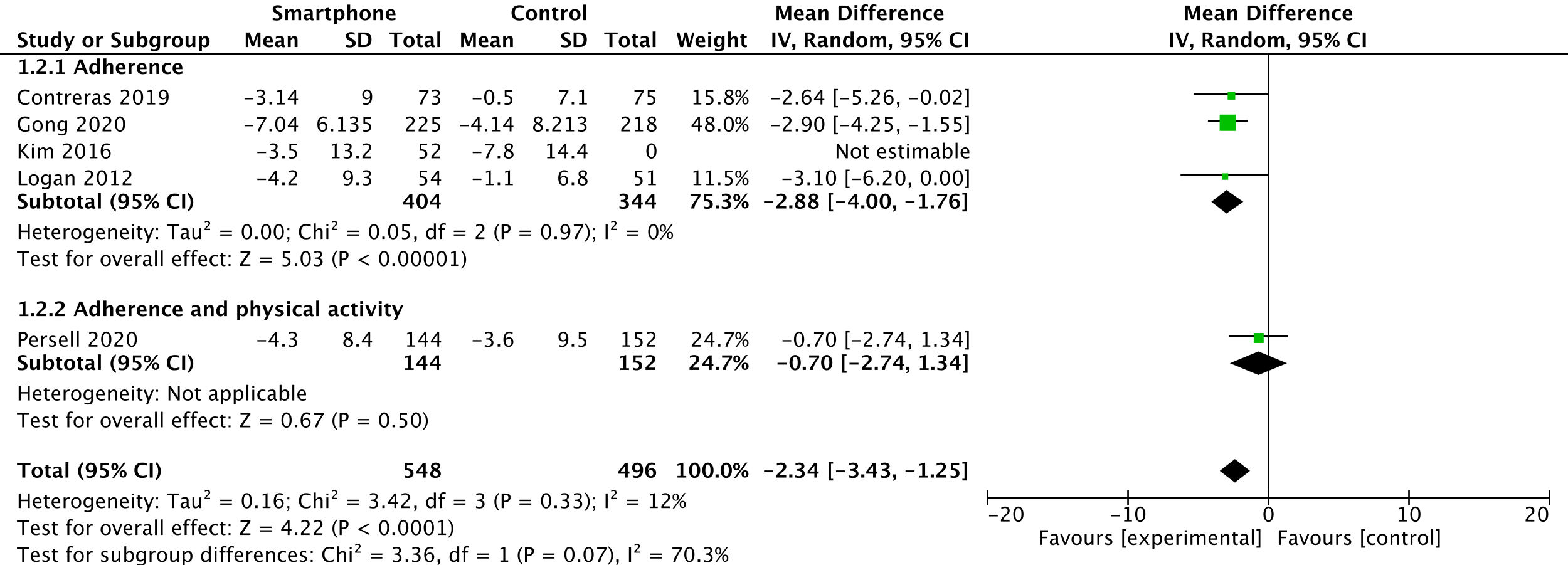

Supplement: Multimedia Appendix 1 [file mhealth_v8i10e21759_app1.docx]
